# Oral health-related conditions in Ecuador: A temporal trend analysis of hospital discharges from 2000–2023

**DOI:** 10.1371/journal.pone.0317440

**Published:** 2025-01-27

**Authors:** Verónica Baldeón, Adriana Hernández, Sthephany Tapia, Alejandro Rodriguez

**Affiliations:** Escuela de Odontología, Universidad Internacional del Ecuador, Quito, Ecuador; Universidade dos Açores Departamento de Biologia: Universidade dos Acores Departamento de Biologia, PORTUGAL

## Abstract

**Background:**

Monitoring hospitalization rates associated with oral health conditions is an important part of epidemiological surveillance, especially when these conditions have increased significantly in low—and middle-income countries. This study aimed to evaluate the temporal trends in hospital discharges associated with oral health-related conditions in Ecuador from 2000 to 2023 and identify the leading diagnoses groups.

**Methods:**

An ecological time-series study was conducted based on annual data from the National Institute of Statistics and Censuses of Ecuador. We identified oral conditions using hospital discharge records, which were classified according to the International Classification of Diseases. We estimated crude and age-standardized rates per 100,000 inhabitants for the entire population and crude rates by sex, age, region, and the diagnosis. Joinpoint analysis was used to identify national trends in hospital discharges.

**Results:**

A total of 93652 hospitalizations were identified. Malignant tumors of the head, face, and neck were the most common diagnosis attributed to hospital discharges (16.5%), followed by cleft palate (14.5%) and cleft lip (7.8%) and other diseases of the jaws (5.4%). The crude rate of OHRC increased from 17.94 to 28.81 hospitalizations per 100,000 population between 2000 and 2023. Based on joinpoint analysis, hospital discharges increased by 2.2% annually during the study period. However, three temporal trends were identified: from 2000 to 2017, hospital discharges increased annually by 4.3% (p<0.05); from 2017 to 2020 decreased by 17.2%; and from 2020 to 2023 increased annually by 12.9%. Average hospitalization rates were higher among those aged 0–9 and > 64 years.

**Conclusions:**

Hospitalization rates associated with oral health-related conditions in Ecuador have increased significantly over the past twenty-four years, except during the COVID-19 pandemic, where cases dropped considerably. Many of the oral conditions identified in the study were malignancies, which are caused by a complex relationship between genetic, environmental, and behavioral factors. Conducting early detection analysis is essential to reduce their occurrence.

## Introduction

Oral Health-Related Conditions (OHRC) have become a significant public health problem worldwide, affecting populations of all ages [1]. Although many of these disorders are preventable through population health interventions, these disorders have increased significantly in recent years [2]. This increase has challenged health systems, especially in low and middle-income countries, where access to dental care is limited in rural areas due to a low proportion of dentists and a lack of oral health programs [1].

OHRC ranges from mild pain dental problems to more complex issues such as orofacial pain, facial disfigurements, and oral cancer, to cite some examples [3]. Among their main risk factors are the consumption of sugars, habits such as alcoholism, smoking, poor oral hygiene, and genetic, environmental, and social factors [4,5]. Additionally, OHRC treatment presents economic and cultural limitations, as well as fears and attitudes of society and health professionals that prevent timely care [6].

Several epidemiological studies have shown that hospitalization rates for preventable dental diseases have increased in the last two decades in low, middle, and even high-income countries [2,7–9]. A study in Brazil, for example, showed an increasing trend in hospital admissions in all regions throughout the years, with most hospital admissions being among older people. The study also found that the leading cause of hospitalizations was cleft lip, oral cavity, and pharynx malignant tumors [7]. Another study in Australia evaluating hospitalization rates from 1998 to 2019 found an increase in OHRC in the country, with dental caries being the most common condition [8]. Another study in New Zealand showed that hospitalization rates for dental diseases increased from 0.92 per 1000 population in 1990–1994 to 2.15 per 1000 population in 2005–2009 [9].

Monitoring hospitalization rates is an integral part of epidemiological surveillance. In the case of OHRC, the monitoring process helps to identify the most relevant health events and disease patterns over time and visualize potentially preventable hospitalization related to dental conditions [10]. Additionally, hospitalization discharges are increasingly used internationally to indicate access to primary care and its effectiveness and to measure the potential health gains from primary care interventions [11]. Unfortunately, OHRC has been poorly monitored in most Latin American countries, and few studies have described their temporal trends [7,12].

In Ecuador, the national registry system of health indicators has improved significantly in the last two decades. However, few studies have used national databases to address topics related to oral conditions [13,14]. As far as we know, only one study has used national databases to evaluate the trend in mortality from oral cancer in the country [13]. This study showed that mortality due to oral cancer in Ecuador significantly increased from 2001 to 2016, with the young groups being the most affected. Nonetheless, the evaluation of hospital discharges on OHRC has been minimally explored. Studying the distribution of hospital discharges related to oral diseases would allow us to understand better the patterns of oral health morbidity and guide effective care and prevention strategies. For this reason, the present study aimed to evaluate temporal trends in hospital discharges associated with OHRC in Ecuador from 2000 to 2023 and identify the leading diagnosis groups.

## Methods

### Study design

We conducted an ecological time series study to evaluate trends in hospital discharge rates related to OHRC in the Ecuadorian population. The unit of analysis was the country from 2000 to 2023. The analysis used secondary data from the National Institute of Statistics and Censuses of Ecuador (INEC).

### Population and study area

The study population was based on all hospital records registered in Ecuador’s public health system over 24 years. Ecuador is an upper-middle-income country, with a GDP per capita of US $6,063 in 2021 [15]. The country covers a total of 283,560 km2 and has four different climatic regions: the Andean region that crosses the country from north to south, which is made up of high mountainous terrain reaching an altitude of over 6000 meters, interspersed by subtropical and temperate valleys; The Amazon region, which is located in the east of the Andes, is an area mainly of tropical forests; The Coastal region, which is located in the west of the Andes, includes subtropical areas and the Galapagos Islands. It has a population of 17,629,765 million inhabitants in 2022, of which 77.5% self-identify as mestizos (mixture of Spanish and indigenous), 7.7% indigenous, 2.2% white, and 4.8% Afro-Ecuadorian [16]. Quito and Guayaquil are the most significant cities, each with approximately 3 million inhabitants. In Ecuador, 64% live in the urban area and 36% in the rural sector, with a life expectancy of 77 years [14].

Ecuador’s health system is fragmented, comprising institutions funded by the government, social security, and private sectors. Public institutions provide healthcare services to the entire population and are organized into four levels of care. Social security institutions cater exclusively to employees and their families. The private sector includes for-profit entities such as hospitals, clinics, dispensaries, doctor’s offices, pharmacies, and prepaid medicine companies in larger cities [17]. Both public and private hospitals must report health and vital statistics data to the Ministry of Public Health and INEC. Likewise, Oral healthcare in Ecuador is divided into three areas: a private system in which the patient bears treatment costs, social security covered by collective funds through taxes, and a state system covered by the state [17]. However, most of the population attends the private system, especially for minor oral complications that do not require hospitalization.

### Data collection

Data on hospital discharge records were obtained from the INEC (https://www.ecuadorencifras.gob.ec/camas-y-egresos-hospitalarios/) in the Statistical Reports section—Beds and Hospital Discharges of the Ecuadorian population. This section contains the annual databases for hospital discharge records for all causes. INEC collects this information through a registry, which all public or private health institutions with hospitalizations must fill out. Twenty-one files corresponding to each year of the study period were downloaded and merged into a single database. Next, all discharges related to oral health conditions were selected based on the International Code of Diseases ICD-10. Due to many codes for dental diseases, hospital discharges were classified by disease groups, as shown in Table 1 [8]. Annual midyear population estimates, total and stratified according to sex, age, and region, were also obtained from INEC.

**Table 1:** Classification of hospital discharge by groups of oral health-related conditions.

Group	CIE-10 Causes
Malignant tumors of the head, face, and neck.	C000 C001 C002 C003 C004 C005 C006 C009 C01 C020 C021 C022 C023 C024 C029 C030 C031 C039 C040 C041 C049 C050 C051 C052 C059 C060 C061 C062 C069 C070 C080 C081 C089 C109 C310 C319 C410 C411 C430 C440 C462 C760 D030 D040
Cleft palate	Q351 Q353 Q355 Q356 Q357 Q359 Q370 Q371 Q372 Q373 Q374 Q375 Q378 Q379
Cleft lip	Q360 Q361 Q369
Other diseases of the jaws	K100 K101 K102 K103 K108 K109
Dental development disorders and eruption	K000 K001 K002 K003 K004 K005 K006 K007 K008 K009 K010 K011
Salivary gland diseases	C088 K110 K111 K112 K113 K114 K115 K116 K117 K118 K119
Cellulitis and mouth abscess	K122
Benign tumors of the head. face. and neck	D100 D101 D102 D103 D164 D165 D230
Diseases of the lips and disorders of the oral mucosa and pharynx	C008 C048 C058 C068 C098 C148 C157 C318 D220 K130 K131 K132 K133 K134 K135 K136 K137
Dentofacial anomalies (including malocclusion)	K070 K071 K072 K073 K074 K075 K076 K078 K079

### Statistical analysis

Hospital discharges were analyzed using frequencies, percentages, and average rates for the study period per 100,000 inhabitants. Crude and adjusted annual rates were calculated using the direct standardization method from the World Health Organization. Additionally, yearly crude rates were estimated by sex, age group (0–9; 10–19; 20–64; ≥64), geographical region (Andean, Coast, and Amazon), and group of cause of discharge.

Joinpoint (JP) regression models were employed to assess trends in hospital discharges for the total sample and sex, age, region, and group of cause [18]. This method identifies the year(s) when a change in trend occurs by connecting various segments of different lines on a log scale at "join points." The analysis begins with zero join points (e.g., a straight line). Then, it identifies points where a statistically significant change in the linear trend slope occurred over time, adding these points to the model. The JP method provides the Annual Percent Change (APC) in rates between the trend change points and estimates the Average Annual Percent Change (AAPC) over the entire study period. The APC is tested to determine if it differs from the null hypothesis (e.g., the annual percent change is 0%). In the final model, each join point indicates a statistically significant change in trends (increase or decrease), and an APC describes each trend. When there are no join points (e.g., no changes in trend), the APC is constant and equal to the AAPC [19].

Data analysis and processing were performed using SPSS 24 software (IBM Corp. Released 2016. IBM SPSS Statistics for Windows. Version 24.0. Armonk, NY: IBM Corp) and Joinpoint software (Version 4.8.0.1) from the United States National Cancer Institute Surveillance Research Program was used for JP analysis.

### Ethical considerations

This study was based on anonymised publicly accessible databases from INEC, which are freely available through the website. This type of study is considered a secondary database study; therefore, an ethics committee was not required.

## Results

### General characteristics of hospital discharges

Table 2 shows numbers and crude average annual rates for hospital discharges attributed to OHRC for the total population and by demographic variables. A total of 93,652 hospital discharges were recorded, of which 54.7% were male, 42.6% were children between 0–9 years, and 48.9% inhabited the coast region. The average annual rate of hospitalizations over the 24-year study period was 25.7 (95% CI 22.72 to 27.41) hospitalizations per 100,000 population. Average annual hospitalization rates were highest among children between 0–9 (53.13 per 100,00 population) and people older than 65 years (42.01 per 100,000 population). No statistical differences existed between males and females or among populations by region.

**Table 2:** Number and average annual rates for hospital discharges by oral health-related conditions diseases in Ecuador from 2000 to 2023.

Characteristics	Categories	Hospital discharges n (%)	Average annual rate per 100,000 population (IC 95%)
	Overall	93,652 (100)	25.7 (22.72–27.41)
Sex	Male	51,202 (54.7)	27.51 (24.95–30.07)
	Female	42,450 (45.3)	22.5 (20.45–24.72)
Age group (years)	0–9	39,882 (42.6)	53.13 (48.33–57.93)
	10–19	11,853 (12.7)	15.96 (14.10–17.83)
	20–64	31,976 (34.1)	16.02 (14.16–17.89)
	+65	9,941 (10.6)	42.01 (38.62–45.41)
Region	Coast	45,751 (48.9)	24.57 (21.69–27.44)
	Andean	44,450 (47.5)	26.59 (24.59–28.58)
	Amazon	3,441 (3.7)	17.38 (14.71–20.05)

**Fig 1:**
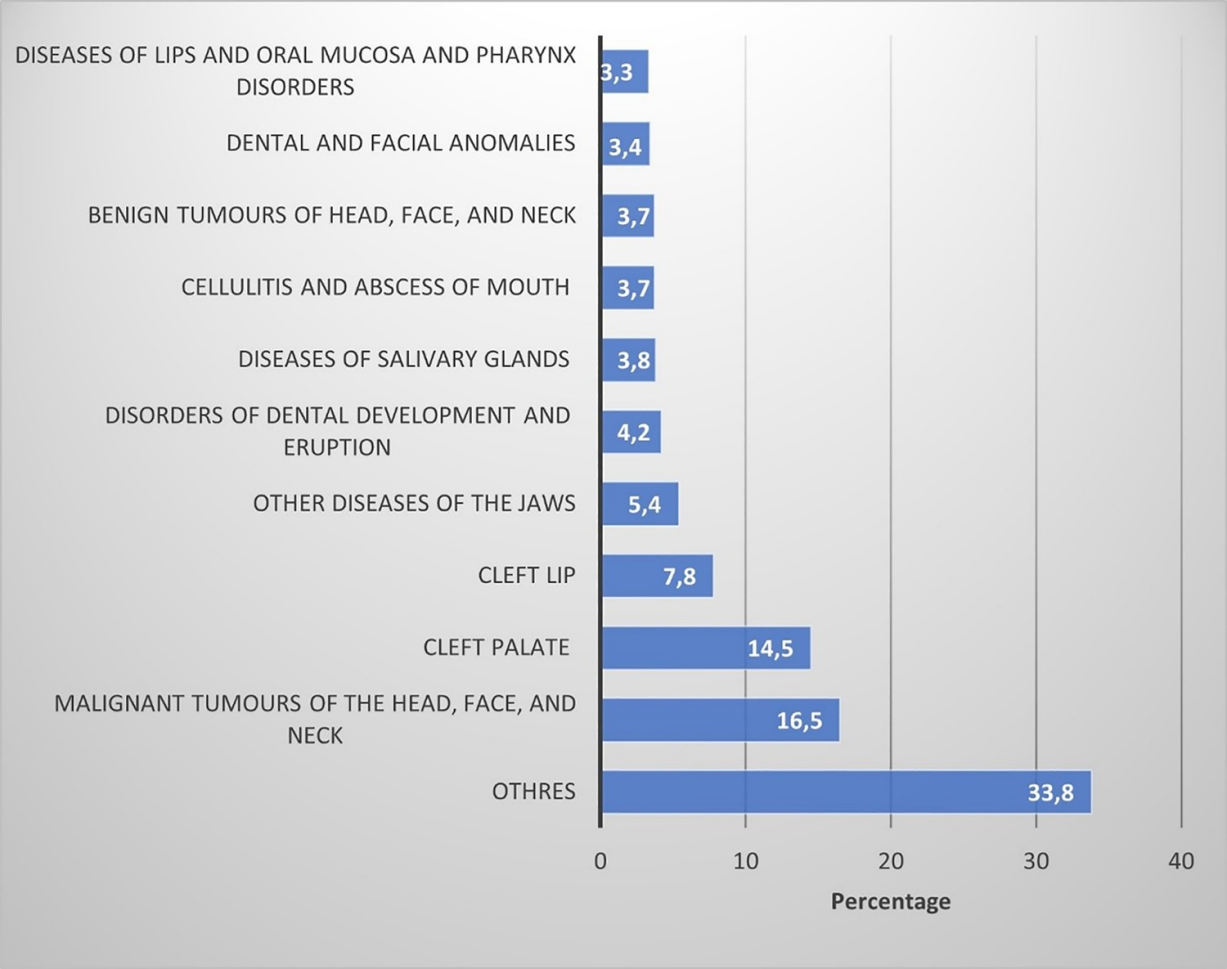
Groups of causes by oral health-related conditions in Ecuador according to hospital discharges, period 2000–2023.

Fig 1 shows the distribution of hospital discharges by OHRC for the entire population. Malignant tumors of the head, face, and neck were the most common diagnosis attributed to hospital discharges (16.5%), followed by cleft palate (14.5%), cleft lip (7.8%), other diseases of the jaws (5.4%), disorders of dental development and eruption (4.2%), diseases of salivary glands (3.8%), cellulitis and abscess of mouth (3.7%), benign tumors of head, face, and neck (3.7%), dental and facial anomalies (3.4%), and diseases of lips and oral mucosa and pharynx disorders (3.3%).

### Trend analysis

Table 3 presents numbers and rates for hospital discharges by sex, age group, and geographical region. Trend analysis in the whole population showed that crude and adjusted hospitalization rates increased. Adjusted hospitalization rates increased from 15.48 to 26.04 hospitalizations per 100,000 population between 2000 and 2019, decreased markedly to 12.71 in 2020, and increased to 28.81 in 2023. The percentual change between 2019 and 2020 in hospital discharges has a reduction of 51%. Similarly, increasing trends were observed for hospital admission rates by sex, age, and region over the study period.

**Table 3:** Crude and adjusted annual rates in hospitalization for oral health-related conditions in Ecuador in the period 2000–2023.

	Total hospitalizations				Rate by Sex		Rate by Age Group				Rate by Region		
Year	N		Crude Rate	ASRs**	Male	Female	0–9	10–19	20–64	< 64	Andean	Coast	Amazon
**2000**	2248		17.94	15.48	19.55	16.32	37.19	10.29	10.75	27.90	21.65	15.11	13.28
**2001**	2289		17.86	15.26	19.31	16.41	37.06	10.64	10.94	27.37	20.71	16.21	9.08
**2002**	2420		18.48	15.97	20.14	16.74	40.65	11.33	9.79	34.08	20.61	17.47	9.95
**2003**	2795		20.98	18.12	23.40	18.45	44.30	14.71	11.65	35.95	22.15	20.59	14.71
**2004**	2889		21.32	19.11	22.83	19.73	44.98	13.93	12.46	36.62	22.65	21.35	9.67
**2005**	2784		20.29	18.07	22.38	18.21	41.35	13.01	12.35	37.15	23.44	18.27	13.09
**2006**	3018		21.61	18.77	23.67	19.57	45.68	13.45	13.09	36.31	24.73	19.13	19.04
**2007**	3012		21.19	18.34	24.15	18.26	45.45	10.73	13.13	41.02	23.52	19.92	13.56
**2008**	3469		23.97	20.38	26.17	21.80	52.52	13.46	14.73	39.62	24.26	24.38	18.42
**2009**	3586		24.33	20.55	26.35	22.34	51.74	15.13	14.79	44.01	26.45	23.43	15.88
**2010**	3838		25.57	21.43	28.20	22.98	55.86	15.29	14.79	45.96	26.73	25.26	19.70
**2011**	4116		26.96	23.20	29.31	24.65	53.75	17.07	17.42	49.35	25.73	27.66	31.84
**2012**	4758		30.66	25.76	33.48	27.88	62.41	20.39	19.38	53.56	31.13	30.61	28.40
**2013**	5040		31.95	27.21	34.80	29.15	60.91	21.70	22.43	51.24	30.65	34.08	24.41
**2014**	5236		32.67	27.47	36.21	29.19	64.40	21.02	23.27	48.66	29.63	36.24	26.37
**2015**	5328		32.73	28.29	36.09	29.43	63.16	22.02	23.56	50.12	30.37	35.63	27.11
**2016**	5236		31.68	27.26	35.21	28.21	64.33	21.05	22.15	47.54	32.71	32.17	20.24
**2017**	5014		29.89	25.61	32.48	27.34	63.36	21.81	19.32	46.72	30.45	31.07	16.03
**2018**	5411		31.79	27.45	34.31	29.32	68.37	20.95	21.07	50.90	34.49	31.20	16.64
**2019**	5199		30.11	26.04	33.10	27.18	68.00	20.17	18.73	50.46	34.65	27.55	17.63
**2020**	2495		14.25	12.71	15.97	12.56	30.02	7.58	10.05	26.84	17.05	12.25	10.10
**2021**	3786		21.33	20.90	23.84	18.87	50.83	12.12	13.79	38.10	25.06	19.21	11.09
**2022**	4571		25.41	25.34	27.92	22.95	59.79	16.46	17.09	41.29	28.93	23.56	14.77
**2023**	5114		28.81	28.28	31.59	24.60	69.09	18.97	17.96	47.63	30.48	27.37	16.20

### Joinpoint analysis

**Fig 2:**
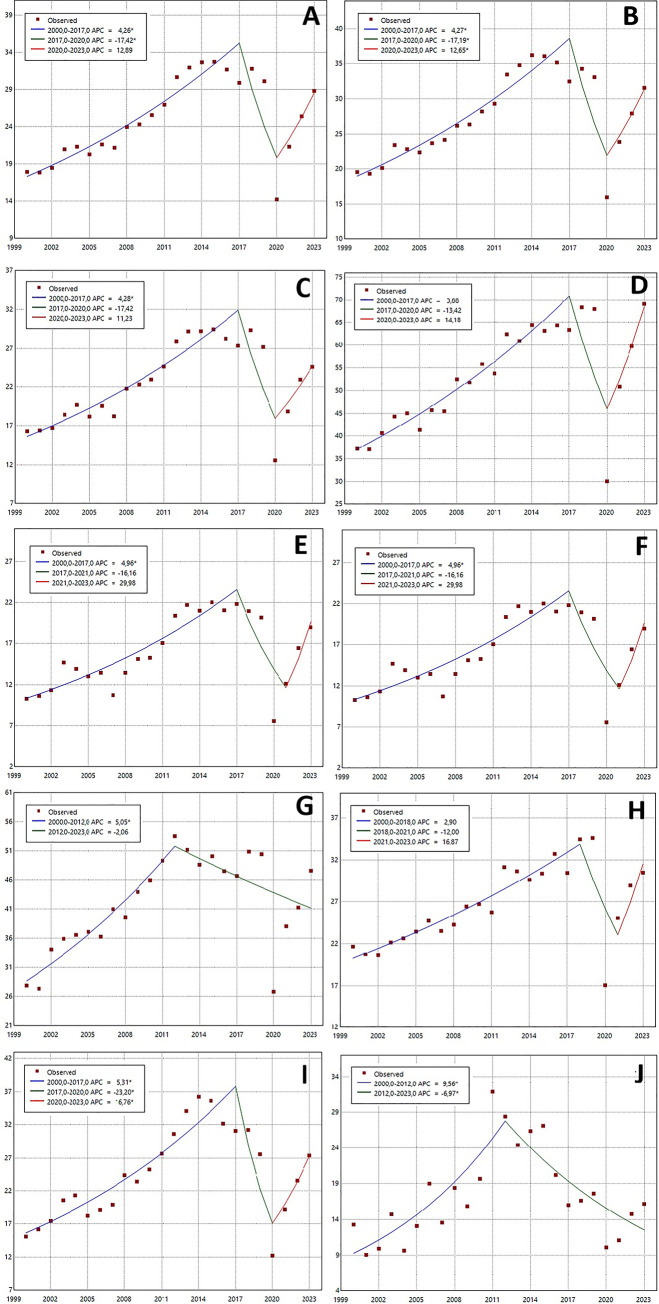
Temporal trends in hospital discharge rates per 100.000 inhabitants for oral diseases in Ecuador, period 2000–2023. The figures represent crude rates per 100.000 for different populations: (A) total population; (B) male population; (C) female population; (D) population of 0–9 years; (E) population of 10–19 years; (F) population of 20–64 years; (G) population over 64 years; (H) population of the Andean; (I) population of the Coast; and (J) population of the Amazon.

Fig 2 and Table 4 show the joinpoint analysis for crude hospital discharge rates as a total population and by sex, age group, and region. Between 2000 and 2023, AAPC in hospital discharges increased significantly by 2.2% annually for the entire population. Similarity increases were identified by sex, age group, and region. Three temporal trends were identified for the total population (Fig 2A). From 2000 to 2017, hospital discharges increased annually by 4.3% (p<0.05); from 2017 to 2020, they decreased by 17.4% (p<0.05), and between 2020 and 2023, they increased annually by 12.9% (p> 0.05). Similarly, three trends were identified for rates by sex (Fig 2B). Hospital discharges for men increased by 4.3% (p< 0.05) between 2000–2017, decreased by 17.2% (p< 0.05) between 2017–2020, and increased by 12.6% (p<0.05) between 2020–2023. Hospital discharges for women increased by 4.3% (p < 0.05) between 2000–2017, decreased by 17.4% between 2017–2020, and increased by 11.2% between 2020–2023 (Fig 2C). By age, hospital discharge for children aged 0–9 increased annually by 3.9% between 2000–2017, decreased by 13.4% (p<0.05) between 2017–2020, and increased by 14.2% between 2020–2023 (Fig 2D).

**Table 4:** Joinpoint analysis for hospital discharge rates for oral diseases in Ecuador, period 2000–2023.

	Period 2000–2023	Tendency 1		Tendency 2		Tendency 3	
	AAPC (IC 95%)	Years	APC (95% CI)	Years	APC (95% CI)	Years	APC (95% CI)
**Overall**	2.2* (1.2 to 3.2)	2000–2017	4.3* (3.2 to 5.7)	2017–2020	-17.4* (-22.8 to 5.2)	2020–2023	12.9 (-0.0 to -33.8)
**Sex**							
Men	2.2* (1.2 to 3.2)	2000–2017	4.3* (3.2 to 5.7)	2017–2020	-17.2*(-22.4 to -5.2)	2020–2023	12.6*(0.2 to 32.9)
Women	2.0* (0.8 to 3.1)	2000–2017	4.3* (2.8 to 5.9)	2017–2020	-17.4 (-23.2 to 4.9)	2020–2023	11.2 (-3.8 to 32.5)
**Age**							
0–9	2.7* (1.1 to 3.9)	2000–2017	3.9 (-2.6 to 6.4)	2017–2020	-13.4* (-20 to 12.5)	2020–2023	14.2 (-2.7 to 38.6)
10–19	2.8* (0.8 to 4.2)	2000–2017	5* (1.6 to 7.6)	2017–2021	-16.2 (-29.6 to 9.2)	2021–2023	30 (-7 to 62.5)
20–64	2.9* (1.6 to 4)	2000–2016	6.1* (4.4 to 8.2)	2016–2020	-13.6 (-22.2 to 6.3)	2020–2023	10 (-4.3 to 29.8)
+64	1.6* (0.0 to 4.1)	2000–2012	5* (2.4 to 19)	2012–2023	-2.1 (-8.5 to 0.2)		
**Region**							
Andean	1.9* (0.2 to 3.3)	2000–2018	2.9 (-8.8 to 28.2)	2018–2021	-12 (-20 to 17.1)	2021–2023	16.9 (-7.4 to 37.8)
Coast	2.4* (1.3 to 3.6)	2000–2017	5.3* (4.2 a 7.0)	2017–2020	-23.2* (-29.6 to -10.7)	2020–2023	16.8*(2.7 to 45.1)
Amazon	1.3 (-0.8 to 4.3)	2000–2012	9.6* (5.3 a 19.8)	2012–2023	-7* (-12.8 to -3.5)		

Hospital discharge for the population aged 10–19 increased annually by 5% (p<0.05) between 2000–2017, decreased by 16.2% between 2017–2021 and increased by 30% between 2021–2023 (Fig 2E). Hospital discharge for the population aged 20–64 increased annually by 6.1% (p< 0.05) between 2000–2016, decreased by 13.6% between 2016–2020 and increased by 10% between 2020–2023 (Fig 2F). Hospital discharge for the population higher than 64 years increased annually by 5% (p<0.05) between 2000–2012 and decreased by 2.1% between 2012–2023 (Fig 2G). Finally, several temporal trends were identified for each geographical region (Fig 2H–2J). Hospital discharge for the Andean population increased annually by 2.9% between 2000–2018, decreased by 12% between 2018–2021, and increased by 16.9% between 2021–2023. Hospital discharge for the Coast population increased annually by 5.3% (p<0.05) between 2000–2017, decreased by 23.2% (p< 0.05) between 2017–2020, and increased by 16.8% (p< 0.05) between 2020–2023. Hospital discharge for the Amazon population increased annually by 9.6% (p<0.05) between 2000–2012 and decreased by 7% (p< 0.05) between 2012–2023.

**Fig 3:**
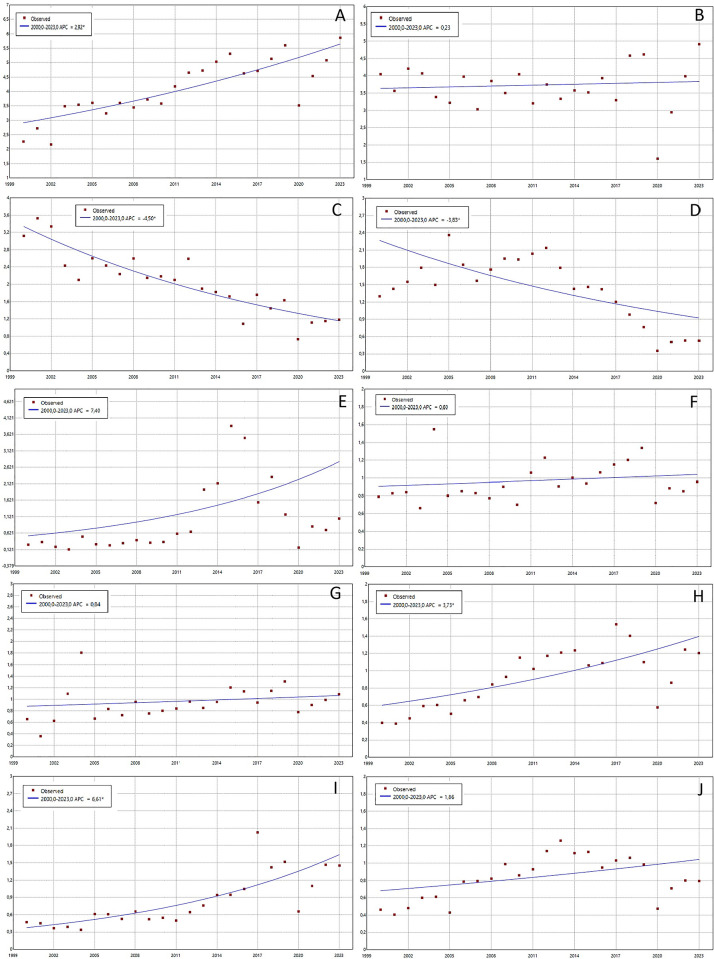
Temporal trends in hospital discharge rates per 100.000 inhabitants by group of causes of oral diseases in Ecuador before the pandemic, period 2000–2023. The figures represent crude rates per 100.000 inhabitants for different groups of causes of oral groups: (A) Malignant tumors of the head, face and neck; (B) Cleft palate; (C) Cleft lip; (D) Other diseases of the jaws; (E) Dental development disorders and eruption; (F) Salivary gland diseases; (G) Cellulitis and mouth abscess; (H) Benign tumors of the head, face and neck; (I) Dentofacial anomalies (including malocclusion); (J) Diseases of the lips and disorders of the oral mucosa and pharynx.

Fig 3. presents the temporal trends for crude rates for Ecuador’s ten most common groups of oral conditions. Malignant tumors of the head, face, and neck increased annually by 2.92% (p<0.05) (Fig 3A); dental development disorders and eruption increased by 7.4% annually (p<0.05) (Fig 3E); Benign tumours of the head, face and neck increased by 3.73% annually (p<0.05) (Fig 3H); and dentofacial anomalies increased by 6.61% annually (p<0.05) (Fig 3I). Conversely, cleft lip decreased by 4.5% annually (p<0.05) (Fig 3C), and other diseases of the jaws decreased by 3.83% annually (p<0.05).

## Discussion

In the present study, we conducted a time series analysis to evaluate the trends in hospital discharges by OHRC in Ecuador over the last twenty-four years. Our results showed that the rate of hospital discharges steadily increased for the total population and subgroups by sex, age, and region. This analysis showed how these trends were affected by the COVID-19 pandemic, where hospital discharge rates fell significantly in 2020. The study also found that, for the entire period (2000–2023), the average rates of hospital discharges were higher for the male population, the population aged 0–9 and >64 years, and the population residing in the Andean region. Additionally, the most common group causes for hospitalizations were malignant tumors of the head, face, and neck, cleft palate, and cleft lip.

As far as we know, our study is the first analysis using a national database of hospital discharges to explore and describe the situation of OHRC in the country. This is important because it allows us to know data on the morbidities treated at the hospital level, information that is related to the population’s burden of oral diseases in the country. Additionally, evaluating hospitalizations helps us identify potentially avoidable conditions that must be treated with appropriate preventive care and early disease management in primary healthcare environments [20].

Our data showed an increasing trend in hospital discharges for OHRC in the country in the last two decades, trends that vary significantly by age group. Similar trends using hospital discharges have been observed in some countries [2,7–9]. However, other countries showed a decreasing trend in specific oral conditions, especially in high-income countries. For example, in the United States, oral conditions such as caries severity, periodontal disease, and tooth loss have declined to historical levels [21]. Likewise, a study conducted in Switzerland showed a decreasing trend in oral conditions in the last two decades [22].

The jointpoint analysis showed that hospitalization rates increased significantly from 2000 to 2017, a rise that slowed down in the next few years and dropped drastically in 2020. These trends could be mainly explained by several socioeconomic and demographic factors rather than an increase in oral conditions. Firstly, the demand for health services is associated with population growth. In the case of Ecuador, between 2000 and 2020. the population increased from 12.531.210 to 17.510.643 [23]. Likewise, between 2006–2017, the number of medical consultations increased from 14.372.251 to 66.899.675, representing a 365% increase [24]. Secondly, over the last two decades, policies and reforms to increase the population’s social and health security coverage have led to a greater demand for medical care in the country [25]. This opening in access to health care increased the number of hospital admissions, including hospitalizations for OHRC. Finally, coupled with social reforms, the country also experienced significant investments in health infrastructure. For example, between 2009 and 2015, 47 hospitals and 74 health centers were built or repaired [24]. This increase in health infrastructure and population growth also affected the number of health workers related to oral health. According to INEC data, the national number of dentists has increased from 1513 in 2000 to 5027 in 2020 [26], influencing the number of patients and hospitalizations for OHRC. However, some of the increases in oral conditions are likely due to an actual rise in specific oral pathologies (e.g., cavities, periodontal disease, etc.), especially those related to changes in lifestyle (alcohol consumption, smoking, poor diet, socioeconomic and demographic factors, etc.) [27].

Since 2015, hospitalization rates have declined, reaching their deepest point in 2020 due to the COVID-19 pandemic. The slight reduction between 2015 and 2019 could be explained by socioeconomic processes, especially those related to the national economy. Since 2016, Ecuador’s economy has declined; therefore, social and health investments have been reduced, directly impacting the population’s access to health. In 2020, due to the COVID-19 pandemic, there were many restrictions on entry to healthcare institutions, causing a marked decline in hospital discharges for almost all diseases. In the case of oral conditions, the pandemic’s impact on hospital discharges was probably more significant than other diseases. This could be explained by the fact that most oral procedures involve direct contact with the patient’s saliva, a means of contagion for COVID-19 [28]. Several studies have documented the effects of the COVID-19 pandemic on healthcare providers and the consequent reduction in visits to such providers [29].

Our study showed that the first group of causes of hospital discharges related to oral conditions in the country were malignant tumors of the head, face, and neck, presenting an increasing trend from 2000 to 2023. Similar trends have been identified in several countries. For example, a study in Mexico found an increase in cases of malignant oral tumors, especially in the population of young adults under 45 years [30]. Likewise, a study in Brazil found an increasing trend of 9% per year from 2006 to 2013 [31]. In Ecuador, a study published in 2018 described the temporal trends of oral cancer from 2000 to 2016. The study concluded that mortality due to oral cancer in Ecuador increased significantly over the 16-year study period, with the young groups being the most affected [13]. However, there is evidence that oral cancer incidence and mortality rates have decreased or stabilized in other countries in the past decades [32]. A possible explanation for the increase in cases of malignant tumors of the head, face, and neck in the country could be associated mainly with the aging of the Ecuadorian population, better diagnosis, and changes in lifestyle such as alcohol and tobacco consumption habits, inadequate diet, sedentary lifestyle, and certain sexual behaviors in young people [33].

Other causes that significantly increased the hospitalization rate were dental development disorders and eruption, benign tumors of the head, face, and neck, dentofacial anomalies (including malocclusion), diseases of the lips, and disorders of the oral mucosa and pharynx. On the contrary, cleft lip presented a significant reduction in the hospitalization rate. In the specific case of cleft lip, the reduction of this condition could be related to health policies as preventive measures that have been implemented in pregnant women in the last decade, such as performing their first control in the first three months and taking medication such as folic acid since it reduces 1/3 the probability of the fetus suffering from cleft lip and cleft palate [34]. Each of these causes must be studied independently to identify the specific factors associated with their trends. Our study is only the first analysis in a series of evaluations that need to be carried out to understand better the distribution of hospitalisations by OHRC in the country.

In some studies, cavities have been mentioned as the leading cause of hospitalisations related to dental diseases [8]. However, in our research, cavities are not among the most important causes of hospital discharge. In Ecuador, hospital discharges refer to patients admitted to a health facility (public or private) for hospitalization, which they leave after a period. People who go to hospitals for oral diseases do so when they require more complex hospital treatment. Furthermore, although hospitals and health centers count on dentists, most people with dental cavities attend private clinics, which are not required to report cases of caries to the Ministry of Public Health or INEC [14].

It is essential to mention that the increasing number of hospital discharges by OHRC directly impacts the cost of hospitalization, both on the health system (e.g., providers) and household levels. However, the direct and indirect costs of hospitalization due to oral conditions have been poorly studied in the country. This burden can lead to significant expenses for medical consultations, hospital stays, tests, and medications. Beyond these direct medical costs, households may face considerable nonmedical and indirect costs, such as transportation, lodging, food, and lost income due to missed work and caregiver responsibilities, especially in rural areas [3]. Reducing the occurrence of hospital discharges, especially those preventable conditions, will reduce the health expenditure for the state and reduce the cost of healthcare for the population.

Our study presents some limitations. First, hospital discharges are not equivalent to indicators of prevalence or incidence, so we cannot estimate precise epidemiological measures. However, hospital discharges are deeply related to population morbidity and represent, in some way, the burden of disease for OHRC in the country. Additionally, hospital discharge data provide an important basis for determining priorities for prevention, emerging issues and trends in incidence worldwide [35]. Second, although the Ecuadorian registration system for deaths and hospital discharges has improved over the last decade, under-reporting (and under-recognition) remains an issue. However, this is unlikely to account for the observed long-term trends fully. Finally, we did not conduct a deep analysis of each group of causes in the study. However, these analyses will be the next steps of this study in the future.

## Conclusion

This study showed that hospital rates for OHRC have been increasing in Ecuador in the last twenty-four years. Trends of increasing rates were also observed for both sexes, all age groups, and in all regions of the country. The impact of the COVID-19 pandemic on hospital discharges was evident, reducing cases by half in 2020 compared to the past year. In Ecuador, malignant disorders such as malignant tumors of the head, face, and neck were the most common diagnoses attributed to hospital discharges. Early detection studies coupled with risk factor analysis must be conducted in the country to reduce the occurrence of these disorders
